# ﻿Surprising discovery of an enigmatic geometrid in Croatia: *Mirlatiaarcuata*, gen. nov., sp. nov. (Lepidoptera, Geometridae)

**DOI:** 10.3897/zookeys.1183.110163

**Published:** 2023-11-01

**Authors:** Axel Hausmann, Gyula M. László, Toni Mayr, Peter Huemer

**Affiliations:** 1 SNSB-Zoologische Staatssammlung München, Münchhausenstr. 21, D-81247 München, Germany SNSB-Zoologische Staatssammlung München München Germany; 2 12 Rainbow Street, Leominster, Herefordshire HR6 8DQ, UK Unaffiliated Leominster United Kingdom; 3 Egelseestr. 21, A-6800 Feldkirch, Austria Unaffiliated Feldkirch Austria; 4 Tiroler Landesmuseen Betriebsges.m.b.H., Natural History Collections, Krajnc-Str. 1, A-6060 Hall in Tirol, Innsbruck, Austria Tiroler Landesmuseen Betriebsges.m.b.H., Natural History Collections Innsbruck Austria

**Keywords:** Cold-adapted, DNA barcoding, geometer moths, Mediterranean, tympanum

## Abstract

A new monotypic genus of Geometridae, *Mirlatia***gen. nov.**, and a new species, *M.arcuata***sp. nov.**, are described from Croatia. Based on external and genitalia characters, the new genus is tentatively placed in the subfamily Larentiinae. However, the new genus takes a highly isolated position by having unique characters of the tympanum and showing an unusually long pectination of female antennae. Genetic analysis of a fragmented DNA barcode (mtDNA; cytochrome c oxidase 1) did not result in a clear assignation to any geometrid subfamily or tribe. Adults, male and female genitalia, and habitat photos of the type locality of the new species are illustrated.

## ﻿Introduction

In January 2014, the third author of this paper acquired the Lepidoptera collection of Robert Hentscholek, an amateur entomologist from Linz (Austria). In the course of inspecting the collection, a male specimen of a peculiar geometrid moth collected in 1983 in Podgora, Croatia (then Yugoslavia) was detected. The moth clearly differed from every known European geometrid species and could not even be assigned to a known genus. The collector was contacted for further information about the unusual moth, and it became evident that a male and a female specimen of the same species had been donated to the late Kurt Huber (1941–2002) who had allegedly passed the two specimens on to a geometrid specialist at the Insect Fair in Weiden, the identity of whom could not be clarified. Later in the same year, two Geometridae specialists, Peder Skou and the first author of this paper, were contacted by TM for help in identifying the unknown species; however, both failed to find out more about the identity and potential relationships of the Croatian moth.

In 2015, the second author independently and unexpectedly had located a female specimen of the same species collected in 1982 in Podgora by R. Hentscholek in the Rudolf Pinker (1905–1987) collection during a survey in the Natural History Museum, Vienna. This specimen is likely the female mentioned by the collector to have been obtained by Huber and seemingly had ended up in the Pinker collection. As the second author also failed to identify the species, he contacted the first author hoping to get the species identified and, thus, the two parallel discoveries of the same problematic geometrid had been linked together. However, as no Geometridae specialist who had been contacted for advice could even attribute the species to a genus, the moth remained unidentified and the whereabouts of one of the three specimens remained unknown. In 2022, with the aim to revive the identification efforts and attempt to trace the missing second male, the third author contacted the fourth author for an opinion about the taxon. As a result, a team was formed to work on identifying the enigmatic geometrid moth. Although the authors could not trace the second male specimen, both sexes of the taxon were available for detailed examination.

After ruling out the possibility of an accidental introduction of the species (see Discussion) we regarded it as a resident species new to science. Venation and shape of hindwing suggest the placement of the species in the subfamily Larentiinae. However, many unique characters (e.g. the peculiar morphology of the ansa of the tympanum) make this attribution rather tentative. A set of largely distinctive morphological characters and the highly isolated position of the taxon based on a DNA barcode analysis suggest that the species belongs to a new monotypic genus, which is together with its type species described in this present paper: *Mirlatiaarcuata* gen. et sp. nov.

## ﻿Materials and methods

### ﻿Morphological studies

The genitalia were dissected, stained with Chlorazol black (holotype) or Eosin red (paratype) and embedded in Euparal on microscope slides applying standard methods of preparation (Lafontaine and Mikkola 1987). Photos of adults were taken using an Olympus Tough TG-5 camera (holotype) and a Nikon D90 SLR camera equipped with Nikkor AF Macro 60 mm lens (paratype). Genitalia were photographed using an Olympus E-M1 camera on a Leica DM2700M stereomicroscope (holotype) and a Nikon DS-Fi1 digital camera mounted on a Nikon Eclipse 80i compound microscope (paratype).

Wing venation was examined under a Wild M3Z stereomicroscope equipped with a Wild drawing tube type 308700.

### ﻿Genetic studies

DNA barcodes were obtained by removing one mid-leg from the dry holotype specimen. DNA extraction, amplification and sequencing of the “barcode” region of the mitochondrial cytochrome c oxidase I (COI-5P) gene region (658 base pairs) were carried out for the outgroup specimens at the Canadian Centre for DNA Barcoding, Ontario, Canada (CCDB), using standard high through-put protocols (Ivanova et al. 2006). For the holotype of the new species a special NGS protocol for ancient DNA in museum specimens was used at the CCDB (Prosser et al. 2016; D’Ercole et al. 2021).

Analysis of DNA barcodes: sequence divergences within and between species were calculated using the Kimura 2-parameter model (Kimura 1980), using the analytical tools provided by BOLD Systems v. 4 platform (Ratnasingham and Hebert 2007; http://www.boldsystems.org/). Phylogenetic and molecular evolutionary analyses were conducted using MEGA v. 11 (Tamura et al. 2021). All sequences and metadata are accessible in the public dataset DS-MIRLATIA (https://doi.org/10.5883/DS-MIRLATIA).

### ﻿Type label data

Information provided in quotation marks is transcribed verbatim. A new line on the label is denoted with “/”, and a different label with “//”; any additional information is provided in square brackets.

### ﻿Abbreviations

**BOLD** Barcode of Life Data System;

**CBG** Centre for Biodiversity Genomics, Guelph, Canada;

**CCDB** Canadian Centre of DNA Barcoding, Guelph, Canada;

**coll.** collection;

**gen.prp.** genitalia preparation;

**NHMV** The Natural History Museum, Vienna, Austria;

**TLMF**Tiroler Landesmuseum Ferdinandeum, Innsbruck, Austria;

**ZSM**SNSB-Bavarian State Collection of Zoology (Zoologische Staatssammlung München), Munich, Germany.

## ﻿Results

### ﻿Genus Mirlatia gen. nov.

Taxon classificationAnimaliaLepidopteraGeometridae

FAEF9D83-9202-5013-B74B-7BF2455B995F

https://zoobank.org/D12B5E0C-3088-47A3-8949-77EF9BFB0A84

#### Type species.

*Mirlatiaarcuata* sp. nov. (by monotypy).

#### Description

**Figure 1.**
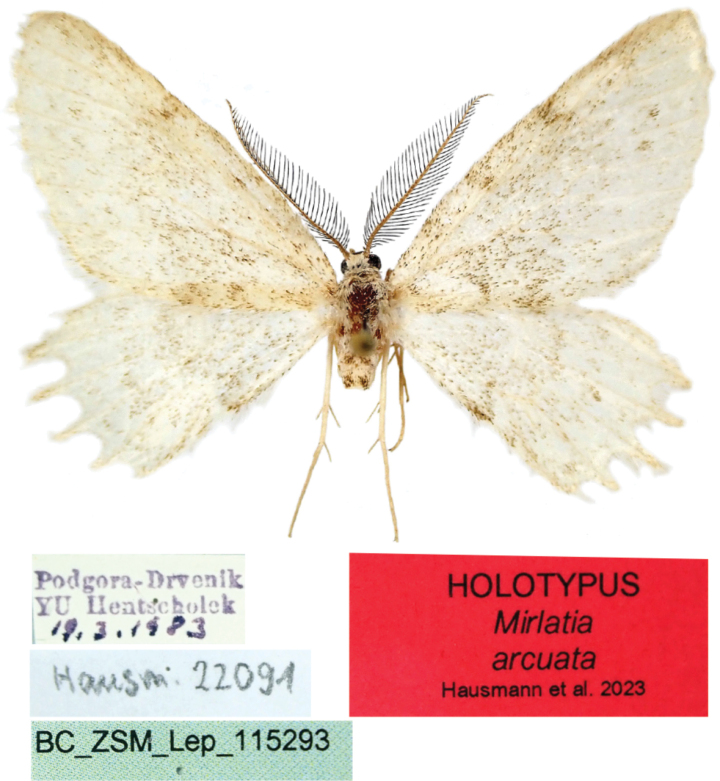
Adult. *Mirlatiaarcuata* sp. nov., holotype, male (coll. TLMF).

**Figure 2.**
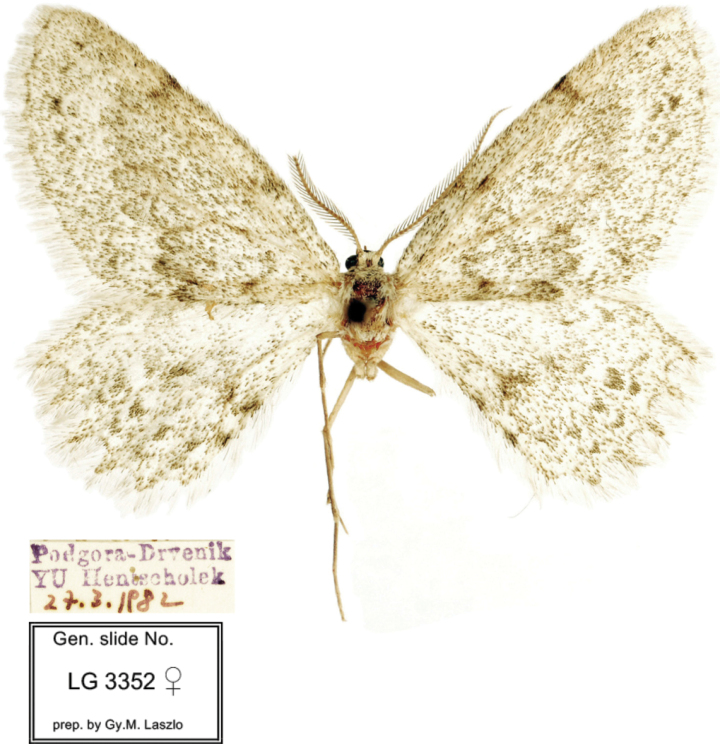
Adult. *Mirlatiaarcuata* sp. nov., paratype, female (coll. NHMV).

**Figure 3.**
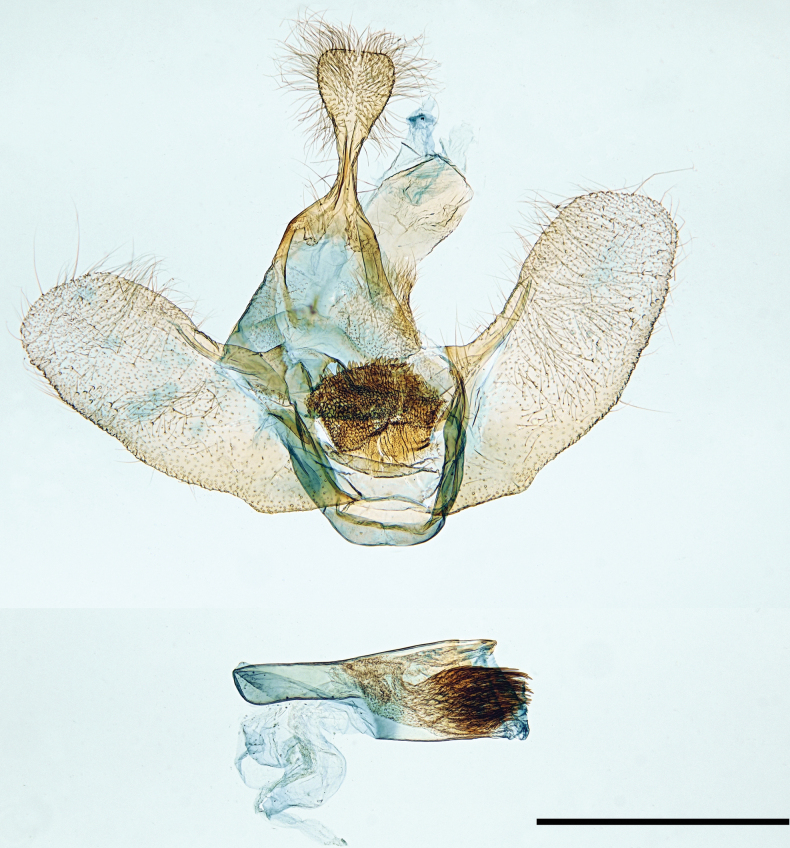
Male genitalia. *Mirlatiaarcuata* sp. nov., holotype, slide No.: G 22091 (prep. A. Hausmann, coll. TLMF). Scale bar: 1 mm.

**Figure 4.**
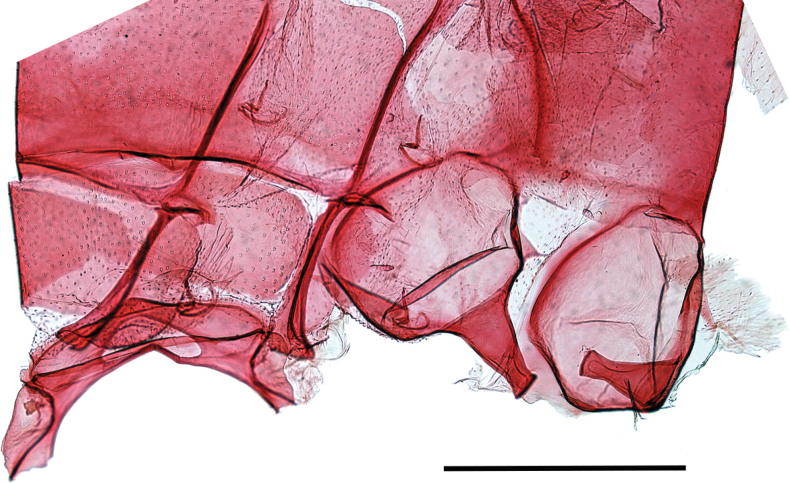
Tympanal organ. *Mirlatiaarcuata* sp. nov., female paratype, slide No.: LG 3352 (prep. G. László, coll. NHMV). Scale bar: 1 mm.

**Figure 5.**
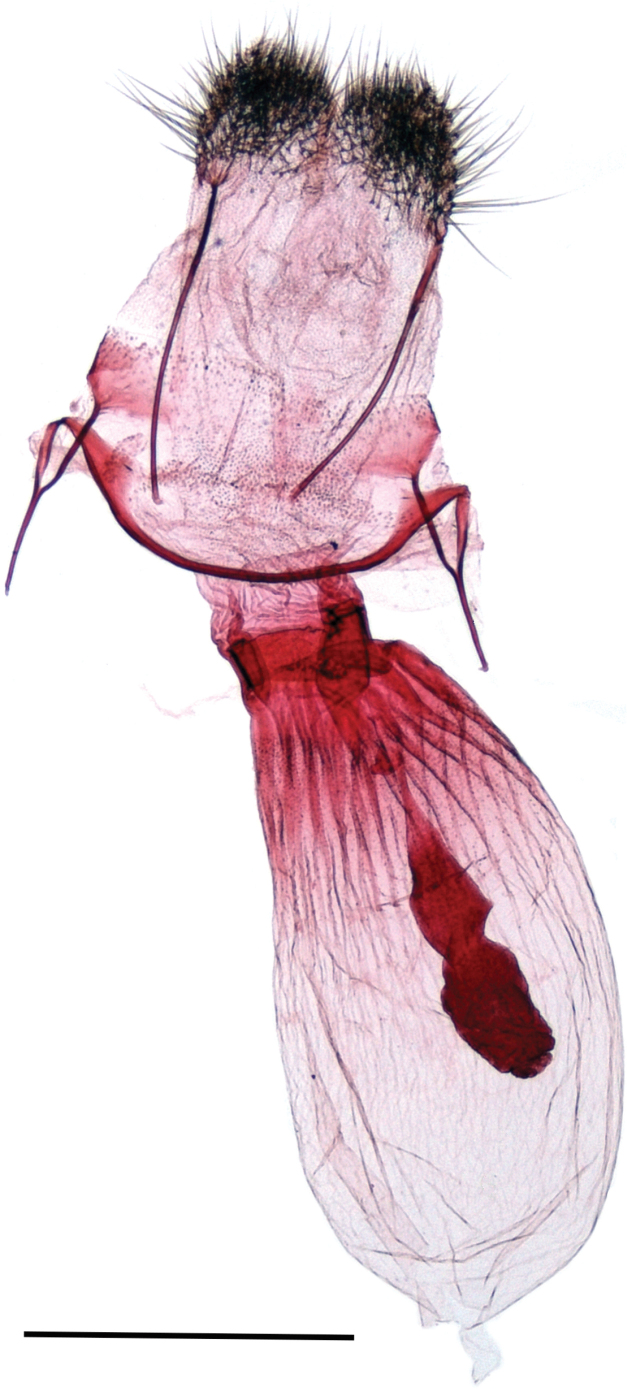
Female genitalia. *Mirlatiaarcuata* sp. nov., paratype slide No.: LG 3352 (prep. G. László, coll. NHMV). Scale bar: 1 mm.

**Figure 6.**
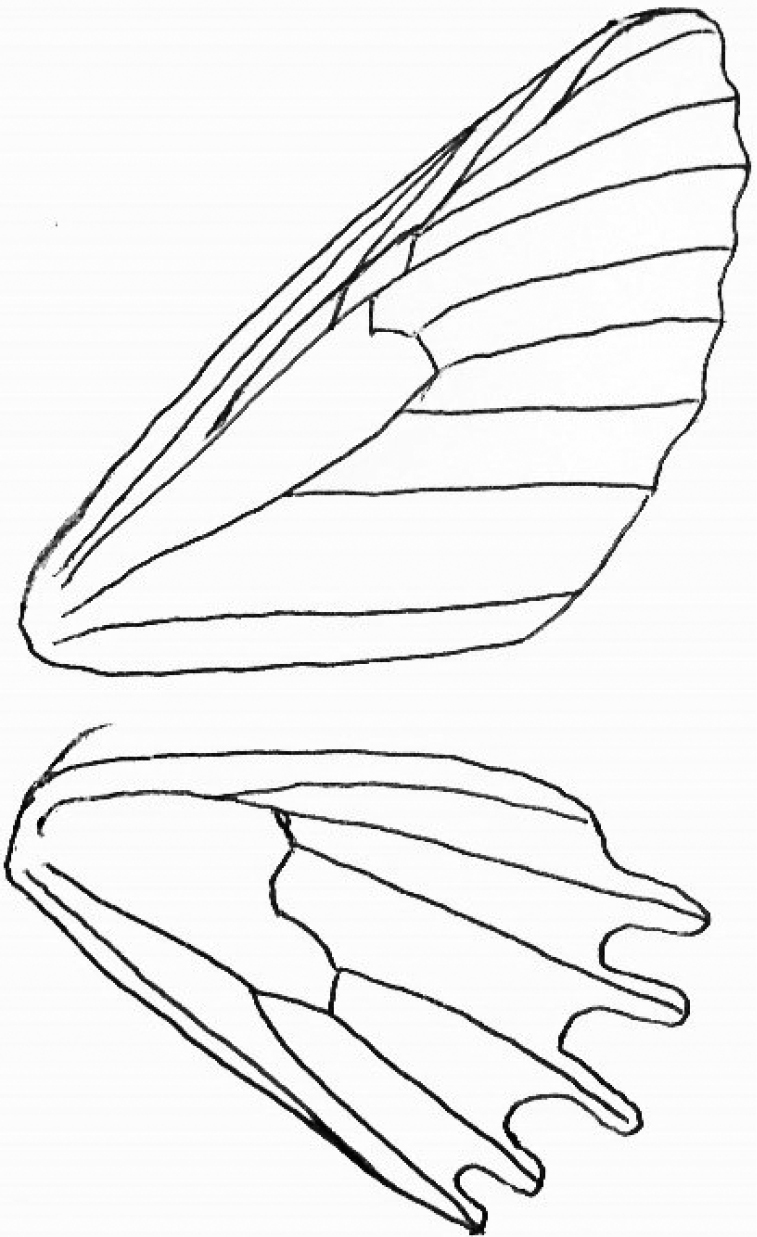
*Mirlatiaarcuata* sp. nov., male, wing venation.

**(Figs 1–6).** External morphology. ***Head*.** Head medium-sized, compound eye relatively large, labial palp narrow, length 1.1× the diameter of eye in male, 1.5× in female. Proboscis well developed. Male antenna distinctly bipectinate, length of longest branches 1.6 mm, approximately 10× the diameter of flagellum, branches checkered black and white; female antenna distinctly bipectinate, longest branches 4–5× the diameter of flagellum. ***Thorax*** (wings, legs). Forewing costa and dorsum slightly, termen markedly convex, apex pointed; hindwing termen distinctly arcuate in both sexes. Transverse lines diffuse, hardly discernible. ***Venation***: forewing with double areole; R2–4 and R5 arising connate from tip of second areole, M1 and R5 shortly stalked laterally on second areole; hindwing with long fusion of Sc+R1 and Rs, distal parts of Rs and M1 completely fused, M2 arising shortly below cell apex (caudad); discocellular cross vein of hindwing distinctly incurved. Male frenulum well developed as a single bristle. Legs long and narrow, spur formula 0-2-4 in male, 0-2-*x* in female (hindlegs of the examined specimen are missing). Male foreleg with long, black, narrow epiphysis arising from middle of tibia.

***Abdomen*.** Tympanal organ (Fig. 4). Ansa of tympanal organ very broad at base, gradually tapered, neither dilated medially, nor at apex with hammer-shaped dilation, tip truncate, with a few micro-spinules in the male.

***Genitalia*.** Descriptions of the male and female genitalia are provided under the description of the type species of the new genus.

#### Differential diagnosis.

Presence of paired tympanal caves with ansa at the base of the abdomen clearly proves the assignation of the new genus to the family Geometridae. *Mirlatia* is distinguished from the vast majority of members of the subfamily Ennominae by the tubular M2 vein of the hindwing and the long fusion of hindwing veins Sc+R1 and Rs. It differs from the genera of the subfamilies Ennominae, Geometrinae, Larentiinae, and Sterrhinae by the very broad base and in the lacking apical dilatation of the tympanal ansa. So far, no other geometrid is known to display a similarly shaped ansa, although a broad ansa basis is found in Archiearinae, Desmobathrinae, and Alsophilini, but these groups have an ansa with a pointed tip. The new genus differs from the vast majority of geometrid genera also by the long pectination of the female antenna.

#### Genetic data, phylogeny.

The genetic data are summarised under the species description. The COI barcode suggests a largely isolated position within Geometridae. The new genus is tentatively placed in the subfamily Larentiinae, supported by the double forewing areole and the hindwing venation with presence of the M2 vein and long fusion of Sc+R1 and Rs. Further aspects of the phylogeny are discussed later in this paper.

#### Etymology.

The new generic name *Mirlatia* is introduced as a feminine noun. It is an aggregate of the stems of two latin words, i.e. *mir*- (stem of the noun *mirum*, *mira* meaning surprise(s)) and *lat*- (*latum*, the perfect participle form of the verb *ferre* meaning to bring, referring to the rather surprising discovery of this curious new geometrid moth.

### ﻿ Mirlatia arcuata sp. nov.

Taxon classificationAnimaliaLepidopteraGeometridae

AD357200-3C0B-5D89-B9EE-6FADB42B2A0E

https://zoobank.org/A451B800-0C1C-47F6-A326-46640D6B7E79

#### Type materials.

***Holotype***: male, “Podgora – Drvenik / YU [southern Croatia, 25 km SE Makarska] [leg. Robert] Hentscholek / [handwritten] 18.3.1983” // DNA barcode sample ID BC_ZSM_Lep_115293 // gen. prp. Hausm. G 22091 // coll. TLMF.

***Paratype***: 1 female, “Podgora – Drvenik / YU [southern Croatia, 25 km SE Makarska] [leg. Robert] Hentscholek / [hand written] 27.3.1982” // [gen. prp. nr. László] 3352 ♀ // coll. NHMV.

#### Description.

For structural external morphology of adults, see the genus description.

**Male** (Fig. 1). ***Wingspan***: 27 mm. ***Colouration*.** Frons brown, vertex whitish. Ground colour of wings whitish, slightly irrorated by brown scales (holotype apparently in a slightly worn state); wing pattern very diffuse, undulate antemedial line and postmedial line marked by brown scales, hardly visible. Ante- and postmedial lines more sharply marked at forewing costa and at inner termen of fore- and hindwing. Discal spots absent on all wings. Fringe concolorous with ground colour.

***Male genitalia*** (Fig. 3). Uncus broadly dilated, spoon-shaped, covered by sparse, long hairs; gnathos absent; juxta with rugose and posteriorly spinulose membrane; saccus short, angular with rounded corners; valva broad, dorsal margin gently concave, ventral margin convex with a short rounded subbasal projection, apex broadly rounded, valva plate largely membranous with narrow costal sclerotization without appendages. Aedeagus stalked in the basal half, abruptly broadened in the distal half; uneverted vesica with fine spinules anteriorly and a large field of aciculate cornuti posteriorly. Sternum A8 and tergum A8 membranous, simple, without appendages.

**Female** (Fig. 2). ***Wingspan***: 29 mm. ***Colouration*.** Frons brown, vertex whitish. Ground colour of wings whitish, heavily irrorated by brown scales; wing pattern diffuse, undulate antemedial line, dentate postmedial line and a few dark brown dots in terminal area vaguely visible on forewing; ante- and postmedial lines more strongly marked at costa and inner termen. On hindwing, dentate medial and postmedial lines more contrasting towards inner termen. Discal spots absent on all wings. Terminal line brown, indistinct. Fringe concolorous with ground colour.

***Female genitalia*** (Fig. 5). Papilla analis broad, evenly rounded, finely setose. Posterior apophysis twice as long as anterior apophysis, the latter ones interconnected by a narrow, strongly sclerotized, evenly arched band. Ostium bursae membranous, corrugated, rather broad. Ductus bursae strongly sclerotized, very short and broad, twice as wide as long. Corpus bursae oval, strongly sclerotized in posterior third, with longitudinal striation towards ductus bursae. One strongly contrasting, elongate structure in the corpus bursae likely referring to remnant of a spermatophore.

#### Genetic data.

The male holotype has been successfully sequenced to a 658-bp fragment containing an internal 200-bp gap. Nearest neighbor: *Idaeamuricata* at a distance of 7.8% in the BOLD barcode-gap analysis, with several next-nearest species at distances of 7.8–8.0% from various families such as Gelechiidae, Blastobasidae, Erebidae, and Euteliidae. Chimera status excluded by examination of both subfragments: Subfragment 1 (3’) with nearest neighbor from genus *Adoxophyes* (Tortricidae: 6.37%) and several geometrids (all subfamilies) at distances of 6.8–7.0%. Subfragment 2 (5’) with nearest neighbor from genus *Ergavia* (Sterrhinae: 5.95%) and several Lepidoptera from other families (e.g. Erebidae) at distances of ~6.2%. Hence, there is no evidence for contamination in both fragments and, therefore, no evidence for a chimera sequence. Nevertheless, from a genetic point of view, our fragmented COI sequence does not allow for a clear assignation of the new genus to any geometrid subfamily, tribe, or genus.

#### Differential diagnosis.

The distinctive features of the new species are discussed under the differential diagnosis of the genus.

#### Etymology.

The species name refers to the arcuate hindwing termen.

#### Ecology, phenology.

**Figure 7.**
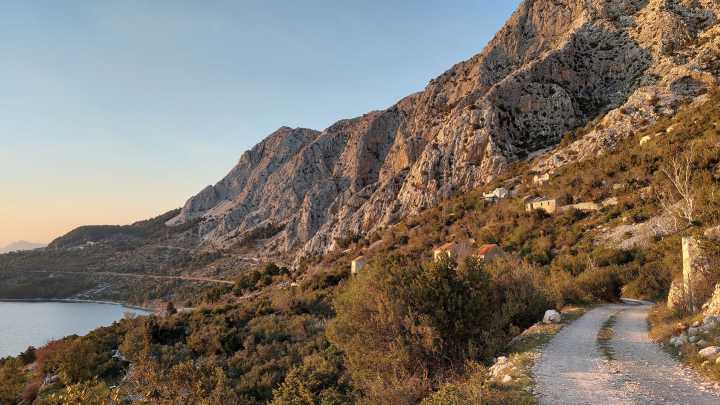
Habitat of *Mirlatiaarcuata* sp. nov., Croatia, Podgora (photograph S. Gomboc).

The type series was collected in southern Croatia at Drvenik (25 km SE of Makarska) from mid- to late March, close to the Mediterranean shoreline, in a habitat dominated by steep limestone rocks and scree (Fig. 7). The worn stage of the holotype indicates that the main flight period might be earlier, i.e. in late winter, and even a hibernation in the adult stage cannot be ruled out. The whitish ground colour of the adults may indicate a possible camouflaging and resting habit among light rocks characteristic at the type locality.

## ﻿Discussion

The phylogenetic position of this new taxon is still unclear and requires further study. Its assignation to Larentiinae is tentative and is mainly based on venation; no larentiine moth is known with a similarly shaped tympanal ansa. The double forewing areole and the long fusion of Sc+R1 and Rs in the hindwing are characteristic for Larentiinae (the latter also present in Alsophilini) (Fig. 6). The distinctly incurved discocellular cross vein is reminiscent of that in Alsophilini (Ennominae), which also possesses a broad-based tympanal ansa. An arcuate hindwing termen occurs in a few species of Larentiinae, e.g. *Triphosadubitata* (Linnaeus, 1758).

The COI barcode data suggests a highly distinct position of *Mirlatiaarcuata* within Geometridae, with the closest species at a large *p*-distance in Sterrhinae (*Idaeamuricata*; in the BOLD barcode-gap analysis). No phylogenetic conclusions can be drawn from *p*-distances of this amplitude; thus, the available data likely point to an isolated phylogenetic position of *M.arcuata*, which is in full agreement with its unique morphological characters. Multi-gene molecular analyses would probably shed more light on the potential relationships of this peculiar taxon and allow to its attribution to one of the described subfamilies and tribes (cf. Murillo-Ramos et al. 2021, 2023), or to recognize it as a member of a distinct new tribe or even subfamily. Nevertheless, these molecular studies could much better be carried out on samples with less degraded DNA. Obtaining fresh samples, however, appears to be a difficult task due to the early flight period and the insufficient knowledge of the habitat preference of the species. Recently, the type locality was investigated for the species during 17–20 March 2022 by Stanislav Gomboc deploying 21 light sources over the course of three nights, but despite this intensive sampling, the species was not captured.

Male and female genitalia of *M.arcuata* sp. nov. do not exhibit any unique characters that would help establish any closer relationships with other geometrid lineages.

As the discovery of a new geometrid in Europe is rather unusual in the third decade of the 21^st^ century, the possibilities of introduction of the species from other continents had to be scrutinised. A possible introduction of this species from other parts of the world can be ruled out from the following reasons:

1. The three specimens collected in March in two consecutive years points to a (at least temporary) reproduction of the species at the site and also suggests that the species is cold-adapted. Furthermore, the species seems to be established in the area, as it was collected in two consecutive years.
2. As no Holarctic species with even similar morphology could be traced by the authors, the only possible region of origin of the species could be in the least known tropics. It is quite unlikely, however, that a cold-adapted species was transported from a tropical country. Nevertheless, the authors thoroughly screened the known geometrids having southernmost distribution in the southern hemisphere (i.e. Chile, Argentina, southern Africa, and southern Australia including Tasmania), where cold-adapted species occur and could not identify any similar species from these regions.
3. The collecting locality at Podgora is not in a direct vicinity of a port, and, additionally, the traffic at Dalmatian harbours during Yugoslavian times was rather limited. Both large ports of Split and Dubrovnik are some 70–80 km away from the locality, a distance that is rather large for a delicately winged early-spring moth with likely limited flying abilities to travel. Additionally, the probability of a tropical species establishing a population and gradually expanding its range in northern Mediterranean areas, where the winter is often cold, is highly unlikely, especially considering the early-spring flying period of this moth. In the early 1980s, under the communist era of the former Yugoslavia, Split or other Croatian ports were rarely visited by ships from other continents.
4. The collector had not been collecting outside of Europe during the time when the specimens were collected, and he had never collected in the tropics at all. Thus, an error in labelling can be excluded.

It may seem peculiar that such a remarkable species has remained undiscovered for so long. However, Dalmatia has not been a highly popular research area for lepidopterists, especially early in the year, in March. It is worth noting that recently there have been other surprising discoveries from the Balkan Peninsula, for example, *Kresnaiabeshkovi* (Ganev, 1987) which belongs to a separate, hitherto unknown genus, and *Idaeamillesima* Hausmann & Prochazka, 2020 (in Hausmann 2020), which is the first *Idaea* species with bipectinate antennae.

## Supplementary Material

XML Treatment for Mirlatia

XML Treatment for Mirlatia arcuata

## ﻿Additional information

### Author ORCIDs

Axel Hausmann https://orcid.org/0000-0002-0358-9928

Peter Huemer https://orcid.org/0000-0002-0630-545X

### Data availability

All of the data that support the findings of this study are available in the main text.
